# Brain Connectivity Variation Topography Associated with Working Memory

**DOI:** 10.1371/journal.pone.0165168

**Published:** 2016-12-08

**Authors:** Xiaofei Ma, Xiaolin Huang, Yun Ge, Yueming Hu, Wei Chen, Aili Liu, Hongxing Liu, Ying Chen, Bin Li, Xinbao Ning

**Affiliations:** 1 School of Electronic Science and Engineering, Nanjing University, Nanjing, Jiangsu Province, China; 2 School of Life Science, Nanjing University, Nanjing, Jiangsu Province, China; University of Toyama, JAPAN

## Abstract

Brain connectivity analysis plays an essential role in the research of working memory that involves complex coordination of various brain regions. In this research, we present a comprehensive view of trans-states brain connectivity variation based on continuous scalp EEG, extending beyond traditional stimuli-lock averaging or restriction to short time scales of hundreds of milliseconds after stimulus onset. The scalp EEG was collected under three conditions: quiet, memory, and control. The only difference between the memory and control conditions was that in the memory condition, subjects made an effort to retain information. We started our investigation with calibrations of Pearson correlation in EEG analysis and then derived two indices, link strength and node connectivity, to make comparisons between different states. Finally, we constructed and studied trans-state brain connectivity variation topography. Comparing memory and control states with quiet state, we found that the beta topography highlights links between T5/T6 and O1/O2, which represents the visual ventral stream, and the gamma topography conveys strengthening of inter-hemisphere links and weakening of intra-hemisphere frontal-posterior links, implying parallel inter-hemisphere coordination combined with sequential intra-hemisphere coordination when subjects are confronted with visual stimuli and a motor task. For comparison between memory and control states, we also found that the node connectivity of T6 stands out in gamma topography, which provides strong proof from scalp EEG for the information binding or relational processing function of the temporal lobe in memory formation. To our knowledge, this is the first time for any method to effectively capture brain connectivity variation associated with working memory from a relatively large scale both in time (from a second to a minute) and in space (from the scalp). The method can track brain activity continuously with minimal manual interruptions; therefore, it has promising potential in applications such as brain computer interfaces and brain training.

## Introduction

Working memory (WM), a core cognitive process, is typically defined as the ability to store and manipulate sensory information over a short period of time on the order of seconds [1]. Although its time span usually lasts for seconds, it comprises multiple processes including sensory perception, information transmission, storage, and retrieval. Therefore, it involves the neural activities of various regions of brain such as the frontal; parietal and posterior cortices; hippocampus; basal ganglia; and cerebellum. [2]. Upon realization that WM emerges from the dynamic interaction and coordination of different brain regions, analyses of correlation, connectivity and network on brain dynamics become necessary. A large body of work has been dedicated to brain connectivity or network research in WM based on various signals through various methods [3–5], e.g., multivariate analysis of functional magnet resonance imaging (fMRI) [6] or diffusion tensor imaging (DTI) [7], coherence, phase synchronization or cross-frequency coupling analysis of magnetoencephalography (MEG) [8] and electrophysiological signals including scalp electroencephalogram (EEG) [9], intracranial EEG or electrocorticography (ECOG) [10, 11], local field potential (LFP) or single unit electrical activity recording [12]. As a direct representation of intrinsic neural electrical activity with high temporal resolution, electrophysiological signals play crucial roles in WM research. To date, there have been great breakthroughs, and it has been reported that various phases of WM are associated with the enhancement of prefrontal-posterior theta coherence [9], an initial enhancement of gamma synchronization followed by a later desynchronization between both medial temporal lobe (MTL) substructures [13], the sustained and stable interareal phase synchrony among frontoparietal and visual areas in multiple frequency bands [14], cross-frequency coupling of the amplitude of high-frequency activity to the phase of slower oscillations in intracranial EEG of the hippocampus [10], and transient theta-gamma phase-synchronization over parieto-occipital regions [15].

To overcome the problem of low signal-to-noise ratios (SNRs) in electrophysiological signals especially scalp EEG, most research needs to discard sections or trials contaminated by artifacts and then apply stimuli-lock average, and/or focus on only a very short period of hundreds of milliseconds after stimuli onset. Thus, the signal became discontinuous and analysis was restricted to a very short time scale. However, brain activity is itself a continual process. In newly developed techniques such as brain-computer interface (BCI) [16] and brain training or cognitive training [17], continuously tracking the subject’s mental activity as it functions in the usual way is quite desirable [17]. Furthermore, the coordination between different brain regions during WM is not necessarily restricted to the time scale of hundreds of milliseconds. Therefore, we try to present a comprehensive view of the brain connectivity associated with WM from a relatively long-term and continuous standpoint, extending beyond the restrictions of stimulus-lock averaging, a short time scale of hundreds of milliseconds after stimulus onset, and manual interruptions such as section discarding.

Our research is based on 16-lead synchronized scalp EEGs collected under three experimental conditions: quiet, control and memory task. The control task was the same as the memory task except that the subjects were not instructed to retain information. Mainly, brain connectivity variations across different conditions were studied. According to previous reports, brain connectivity can be described as structural, functional or effective [3, 4]. In this manuscript, we refer to the functional connectivity that denotes the symmetrical statistical association or dependency between different leads [3]. To study brain connectivity, each EEG lead location is treated as a node, and correlation between signals of two different leads is reflected by a link index. The Pearson correlation is a classic correlation measure. Its application to EEG analysis can be traced back to the 1940s according to [18], and recently, it has been reported that a Pearson correlation based brain connectivity measure has promising potential to identify epileptogenicity and predict the outcome of epilepsy surgery [19]. Nevertheless, reports of brain connectivity associated with WM or other cognitions based on EEG Pearson correlations are limited and negative [20, 21]. After carefully examining Pearson correlation’s applicability to EEG as well as the interpretation, we suggest calibrating its usage in EEG analysis, i.e., calculating Pearson correlation only for matched frequency and interpreting it as correlated or not after comparing with matched surrogates. Following that, we propose two indices: link strength *L*, which is contributed to only by a significant correlation coefficient, and node connectivity *C*, which is defined as the sum of all link strength linked with a node. We then investigate the variations of *L* and *C* among states and build up brain connectivity variation topography. The profound results lie in beta and gamma rhythms; that is, (i) when comparing the memory and control states with the quiet state, the beta topography indicates links strengthening between T5/T6 and O1/O2, which reveals the visual ventral stream [22]; (ii) the gamma topography conveys strengthening of inter-hemisphere links as well as weakening of intra-hemisphere frontal-posterior links and implies parallel inter-hemisphere coordination as well as sequential intra-hemisphere frontal-posterior coordination in visual and motor processing; (iii) when comparing memory with control, the gamma topography shows an increase in T6 node connectivity, which provides strong evidence for the information binding or relational processing function of the temporal lobe in memory forming [2] from scalp EEG. To our knowledge, it is the first time for any method to effectively capture brain connectivity variations associated with working memory from a relatively large scale both in time (from a second to a minute) and in space (from the scalp). This method can track brain activity continuously with minimal manual interruptions, so it has promising potential in applications including brain computer interfaces and brain training.

## Materials and Methods

### Experiments

Fourteen volunteers with college educations or higher, including 13 males and one female, were enrolled in this experiment. On average, the subjects were 24.9±4.8 years of age (mean±SD). Experiments were ethically approved by the institutional review board of the School of Electronic Science and Engineering of Nanjing University, all participants were informed of the research aim as well as the content of experiments and signed informed consents.

During the experiment, the subject was seated in front of a 19’ monitor at a distance of approximate 25 inches, with room for adjustment for comfort and clear sight. Each subject sequentially underwent three different conditions: quiet, control, and memory task.

During the quiet scene, the subject remained motionless and relaxed with their eyes open. The monitor presented nothing but a grey background, so that the subject did not have to fixate. Signals collected under this condition are viewed as baselines.

Just before the control scene began, the subject was informed that pictures would be presented on the screen, and they were instructed to click the mouse randomly when an option button appeared. In fact, this task was presented in the same way as the memory task, which is described in detail in the next paragraph.

Immediately before the memory task began, the subject was told that the scene was meant to test their short-term memory and that they should try their best to retain and recall as the prompted and make the correct choice by clicking the option button. A delay-match-to-sample paradigm [2] was used in the memory task. In this scene, objects were randomly presented as one of three different shapes (round, square and triangle) in three different colors (red, green and blue), resulting in nine different combinations in total. The scene consisted of five sections with 2-s breaks between every two adjacent sections, and each section lasted 10 seconds, including sequentially a 2-s memorization block, a 2-s retention interval, and three successive 2-s test blocks. In the memorization block, the monitor presented one object and simultaneously prompted the subject to memorize it. After the 2-s retention interval, in each test block, the subject was presented with an object and instructed to judge whether it was same as what he had seen in the memorization block and choose the corresponding option (‘yes’ or ‘no’) by clicking the mouse.

**Fig 1:**
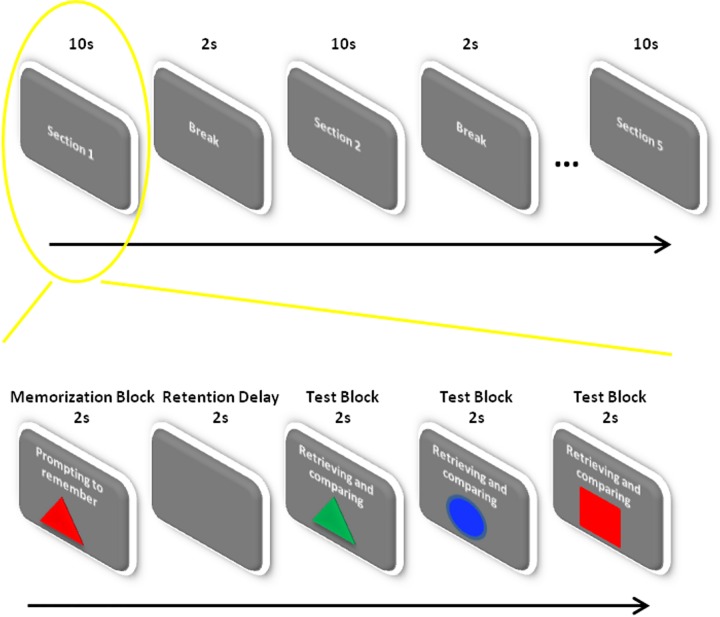
Experimental paradigm in memory and control tasks. The scene consisted of five sections with 2-s breaks between every two adjacent sections, and each section lasted 10 seconds, including a sequential 2-s memorization block, a 2-s retention interval, and three successive 2-s test blocks. In the memorization block, the monitor presented one object and simultaneously prompted the subject to memorize it. After the 2-s retention interval, in each test block, the subject was presented with an object and instructed to judge whether it was same with what he had seen in the memorization block and choose the corresponding option (‘yes’ or ‘no’) by clicking the mouse. The memory task and the control scene were the same except that at the beginning of the memory task, the subject was instructed to make an effort to memorize and make correct answers; for the control tasks, they were not.

The experimental paradigm of memory task and control is illustrated in Fig 1.

**Fig 2:**
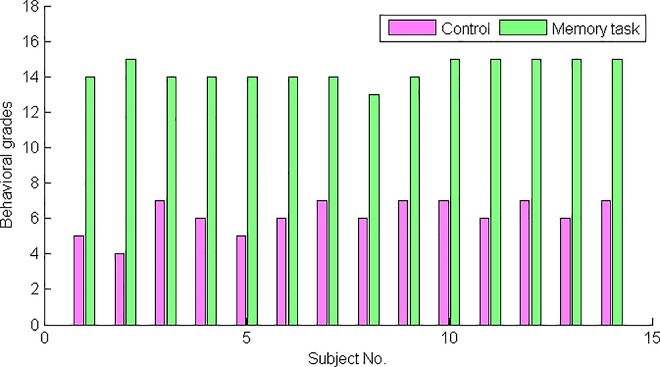
Behavioral grades of all subjects in the control (in magenta) and memory (in green) states. The grade is equal to the number of correct option clicks, with a full score being 15. In the end, all subjects received scores of no less than 13 in the memory task and no more than 8 in the control state.

In all scenes, subjects were required to keep as motionless as possible except for clicking the mouse when necessary. After each scene started, subjects were monitored for physical activity, and all subjects were required to avoid any visually discernible motions except for the slight right hand movement and finger clicking. The subjects’ mental activities were evaluated and recorded by the automatic grading program integrated in the experiment program. The grade is equal to the number of correct option clicks, with a full score being 15. Detailed grades for each subject in the memory and control states are illustrated in Fig 2. In the end, all subjects received scores of no less than 13 in the memory task and no more than 8 in the control state.

### EEG acquisition and preprocessing

The 16-lead scalp EEG of the subjects was recorded by Nihon Kohden EEG-9100. Electrode sites of the international 10–20 system were used for 16 scalp electrodes referring to the ear lobe of the same side. The recording was made with a sampling rate of 500Hz, a time constant of 0.3s and a high cutoff frequency of 120Hz. For each condition, the EEG was recorded continuously for 1 minute.

Considering that EEG is easily contaminated by artifacts, we focused the investigation on the rhythms of frequencies higher than 4Hz. Therefore, most components of electrooculogram (EOG) as well as the low-frequency drifts and bias exerted few effects on this study [23, 24]. In addition, for artifacts with a frequency higher than 4Hz, we applied a statistic regarding its duration percentage to the series duration. Based upon the fact that the neural oscillation amplitude during the waking state is rather small, the sampling points exceeding ±40μV were considered to be severely contaminated by artefacts and of low SNR, as is usually the case in artifact rejection procedures [23, 25]. For each series, we calculated the duration percentage of severely contaminated segments. For most series the percentages are far less than 5%, which is too trivial to distort the result. However, there are exceptional cases, including Fp1 and Fp2 of subject No. 6 in the memory state (both portions are approximately 10%) and T4 of subject No. 13 in the memory and quiet states (24% and 48%, respectively). To guarantee reliability, we discarded these two subjects when calculating the group average.

The original EEG with a length of 1 minute was filtered into four frequency ranges: theta (4-8Hz), alpha (8-13Hz), beta (13-30Hz), and gamma (30-80Hz). After removing the beginning and ending, which were affected by digital filtering, there were no less than 50 seconds remaining. The remaining was then segmented into sections with a length of 1s without overlap.

### Pearson correlation in EEG

The following analysis is based on Pearson correlation. Pearson correlation is a classic statistical measure of linear correlation between two variables. However, one should be very careful when applying it directly to EEG data for several reasons:

First, EEG is not an uncorrelated stochastic signal, which means that an intrinsic temporal structure exists. Therefore, the sample rate will affect the result of the correlation analysis, e.g., with the same sample size, oversampling will exaggerate the correlation, while undersampling will underestimate the correlation. The usual solution to avoid such a bias is by complying the sample rate with the frequency of the signal. However, EEG is not stationary, and its dominant frequency constantly varies from state to state. Under such non-stationary conditions, it is not recommended to treat all components of EEG as a whole when calculating a correlation coefficient. Instead, we calculated correlations in separated rhythms of theta, alpha, beta, and gamma. Undoubtedly EEGs in separated rhythms are investigated for specific reasons in neuroscience, whereas in signal processing, it has the additional significance of making two signals comparable.

The second concern lies in the interpretation of the correlation coefficient. In practical applications, the same configurations, e.g., sample size and sample rate, are usually used regardless of the difference in intrinsic frequency of the different rhythms. Thus, it is not reliable to directly interpret a great coefficient as a high linear correlation. To solve this problem, we utilized the shuffle surrogate method to estimate the thresholds, which were used in the judgment of significant correlations for different rhythms separately.

#### Shuffle surrogate method

First, the original EEGs were randomly shuffled lead by lead independently; next, they were filtered into four rhythms of theta, alpha, beta, and gamma and sectioned into 1s lengths without overlap, then Pearson correlation coefficients between two leads for a specific rhythm were calculated section by section. For each lead or channel, shuffling was conducted independently 100 times in order to obtain a reliable distribution of correlation coefficients. Finally, the 2.5 percentile and 97.5 percentile of coefficients were used as the thresholds of corresponding rhythms between the two leads, and the coefficient *r* calculated from un-shuffled signals was compared with the corresponding threshold of matched rhythms.

### Brain connectivity variation topography

In this research, we try to present a comprehensive description for the relationship between different brain regions on a relatively long time scale, i.e., from 1 second to 1 minute, under the designed experimental conditions. Assuming each EEG lead location to be a node, we first define the link strength between two nodes, then define the node connectivity for each node, and finally construct the brain connectivity variation topography including both nodes and links.

#### Link strength

The link strength between two different nodes A and B is defined as:

$$L_{A,B}=\sum_{r_{{}_{A,B}}^{i}\notin \Phi_{r}{}_{\_surr}}\left|r_{{}_{A,B}}^{i}\right|/\#(r_{{}_{A,B}}^{i})$$ (1)

in which $r_{{}_{A,B}}^{i}$ is the Pearson correlation coefficient calculated between signals derived from node A and B for the *i*th section; Φ_*r*_*surr*_ is the 95% confidence interval of the corresponding surrogates’ correlation coefficients, and # means ‘the number of’. Thus $\#(r_{{}_{\text{A},\text{B}}}^{i})$ also equals the number of sections of analyzed signals. This definition attributes the link strength only to coefficients that fall beyond the 95% confidence interval. As a consequence, a higher *r* will not be considered a stronger correlation than a lower one if it still falls into the 95% confidence interval of corresponding surrogate’s *r* distribution. Theoretically, *L* has the range of [0, 1].

#### Node connectivity

In this manuscript, we define an index of node connectivity to evaluate the node importance as a connection hub. Herein, the connectivity of node A is calculated as the sum of all strengths linked with it; that is,

$$C_{\text{A}}=\sum_{j\neq \text{A}}L_{A,j}$$ (2)

Given the total lead number as 16, *C* theoretically has the range of [0, 15].

#### Topography

Based on the indices of both link strength *L* and node connectivity *C*, we depict the difference between states by constructing brain connectivity variation topography. That is, for each node, we map the node connectivity variations ∆*C* between two states into the color of nodes, and we map link strength variations ∆*L* into the color of links, thus plotting the topography to explore the spatial distribution characteristics of coordination between brain regions concerning specific brain activity. In addition, in order to clearly present the direction of the variation, we applied a solid line style for increasing link strength, a dashed line style for decreasing link strength, a larger font size of lead caption to indicate increasing node connectivity and a smaller font size to indicate decreasing node connectivity.

Finally, there are 120 possible undirected links for 16 leads, and it would be quite confusing to plot them all. Therefore, with a two-sample Kolmogorov-Smirnov (KS) test, only those links with significant differences between states (*p*<0.001) are plotted.

## Results

### Shuffle surrogates’ Pearson correlation coefficient

**Fig 3:**
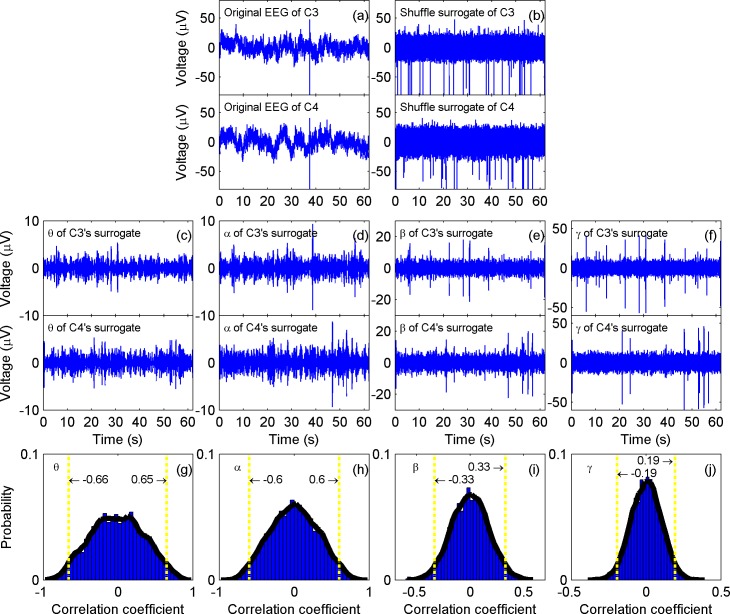
EEG shuffle surrogates’ Pearson correlation. (a) Original EEG of one subject’s C3 and C4 during the memory state. (b)* Shuffled surrogates of (a). (c)-(f) Theta, alpha, beta, and gamma rhythms of (b), respectively. (g)-(j) Histogram of correlation coefficients in surrogates’ theta, alpha, beta and gamma rhythms, respectively. To obtain a reliable distribution as in (g)-(j), the original signals in (a) have been randomly shuffled 100 times independently, and (b) and (c)-(f) only depict one of the implementations. It is shown that distributions of the Pearson correlation coefficients for surrogates are symmetric sub-Gaussian, and the kurtosis increases with increasing frequency. Thus, a greater *r* for the theta rhythm does not necessarily mean a higher correlation than a lesser *r* for the gamma rhythm. The 2.5 percentile and 97.5 percentile, which are marked by vertical yellow dashed-dot lines in (g)-(j), were chosen as thresholds of corresponding rhythm. *: Although it appears that (b) is completely different from (a), especially in that there are seemingly many more spikes in (b), the histograms of (a) and (b) are exactly the same. The rough look of (b) is the result of randomly dispersing the time consecutive samples with relatively high amplitude, which compress together in (a), into different locations.

A typical result of EEG shuffle surrogates’ Pearson correlation is presented in Fig 3.

It was found that the surrogates’ coefficient *r* distributions are symmetric sub-Gaussian, with kurtosis less than 3 and the absolute value of skewness less than 0.05. It was also found that the kurtosis increases with increasing frequency, meaning that a greater *r* for the theta rhythm does not necessarily mean a higher correlation than a lesser *r* for the gamma. In the meantime, although the distributions are different across different rhythms, they remain quite consistent across different EEG pairs as well as different states; that is, the hypothesis that two sample sets are from the same distribution cannot be rejected with two sample KS test *p*>0.3.

### Representative result for one subject

Each subject was examined using the method described in the methods section, and the results of one representative are presented below.

#### Pearson correlation coefficient

**Fig 4:**
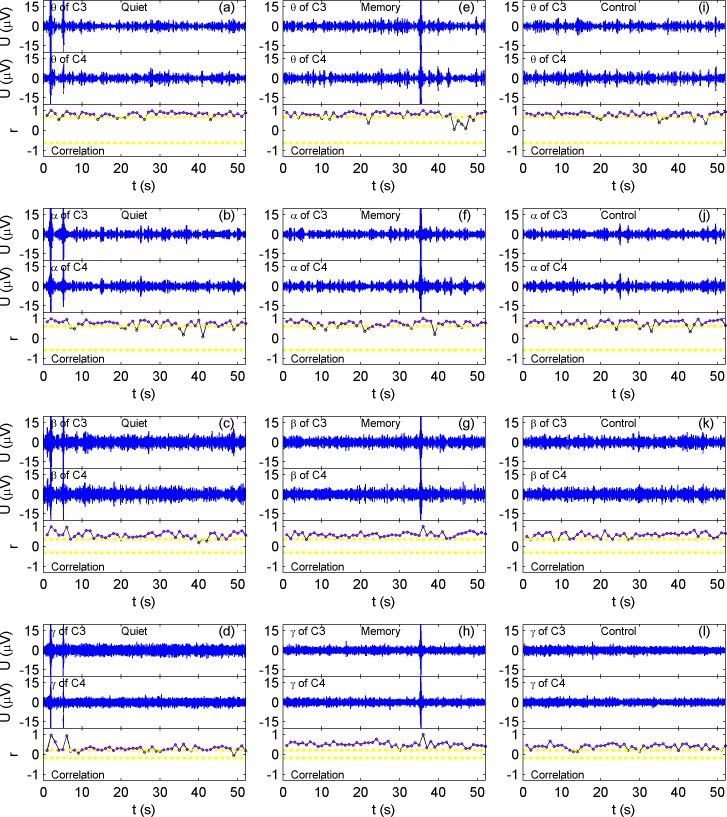
Rhythm profiles of C3 and C4 and their corresponding correlation coefficient fluctuation. There are 12 sub figures, (a)- (i), among which from the left to right column, they are quiet, memory and control states, respectively, and from top to bottom, they are theta, alpha, beta, and gamma, respectively. Within each sub-figure, they are C3 and C4 rhythm plots and correlation coefficient fluctuation from top to bottom, respectively. In the correlation coefficient plots, the preset thresholds are depicted as horizontal yellow dash-dot lines, and dots that fall beyond the thresholds are drawn in magenta. It can be seen that there are significant differences between states for the gamma rhythm.

As a representative, the four rhythms of C3 and C4 of one subject under three different conditions and the correlation coefficient fluctuations are presented in Fig 4. We also applied a two-sample KS test to the correlation coefficients between different states, and the results are presented in Table 1.

**Table 1:** *p* of two-sample KS tests of correlation coefficients in Fig 4.

	Memory vs. Quiet	Control vs. Quiet	Memory vs. Control
**Theta**	0.858	0.858	0.961
**Alpha**	0.997	0.065	0.037
**Beta**	0.262	0.858	0.702
**Gamma**	10^−13^	10^−6^	10^−4^

From Fig 4 and Table 1, it can be seen that in the theta, alpha and beta rhythms, there are no significant differences between every two states (*p* > 0.01). However, in gamma, linear correlations between C3 and C4 are significantly stronger in the memory and control states than in the quiet state.

#### Brain connectivity variation topography

**Fig 5:**
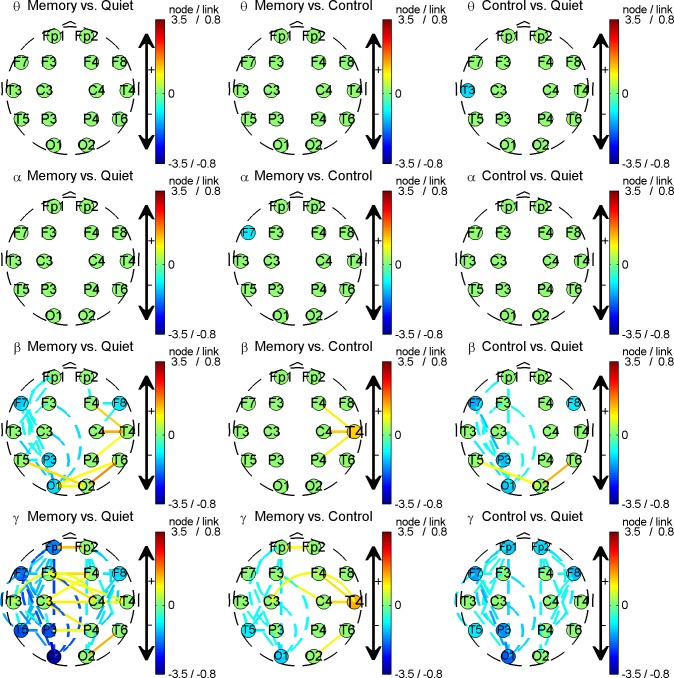
Trans-states brain connectivity variation topography for the same subject as in Fig 4. Each row corresponds to a rhythm, and from top to bottom, they are theta, alpha, beta, and gamma respectively; each column represents a comparison between states, and from left to right, they are memory vs. quiet, memory vs. control, and control vs. quiet respectively. Lines between nodes depict the link strength variation, i.e. ∆*L*, between two states. A dashed line indicates a decreasing link strength and a solid line indicates an increasing link strength. The amplitude and sign of ∆*L* are also mapped into the color of the lines. To avoid confusion caused by drawing all 120 links among 16 leads, only links with significant differences between states, i.e., *p*<0.001 in the two-sample KS test, are depicted. Nodes (round patches) with captions represent the change of node connectivity, i.e., ∆*C*. In detail, a larger caption size means an increase in *C*, and a smaller size means a decrease, while a medium size means no discernible changes. The amplitude and sign of ∆*C* are mapped into the color of the node as well. It is noted that there are certain nodes with a discernible change in *C* while there are no lines connected with them, e.g., T3 in control vs. quiet theta and F7 in memory vs. control alpha. This is no mistake: because we only plot lines for links with statistically significant differences between states while summing all links connected to the node when calculating ∆*C*, the information conveyed by the nodes and lines does not completely overlap.

Based on definition put forth in the methods section, we plotted brain connectivity variation topography of this subject, as presented in Fig 5.

As seen from Fig 5, there is not much difference between the states in the theta and alpha rhythm, while in beta, and more prominently in gamma, there are indeed differences between states:

1. For the beta rhythm, the frontal-posterior link strengths of the left hemisphere decrease, while the O2-T5/T6 link strengths increase for both transitions from quiet to memory and from quiet to control. T4 node connectivity in the memory state is higher than that in the control state.
2. For the gamma rhythm, it is also found that most ipsilateral frontal-posterior links become weaker in control and memory states compared with the quiet state. At the same time, contra lateral links among F3, F4, C3, C4, P3, and P4 are found to be getting stronger when comparing memory state with quiet. Comparing the memory state with the control state, the ipsilateral frontal-posterior link strength of the left hemisphere decreases, and node connectivity *C* of T4 evidently increases.

To ensure the consistency of the above results across subjects, we investigated on a group level and present the interpretation in the following section.

## Results for the group average

**Fig 6:**
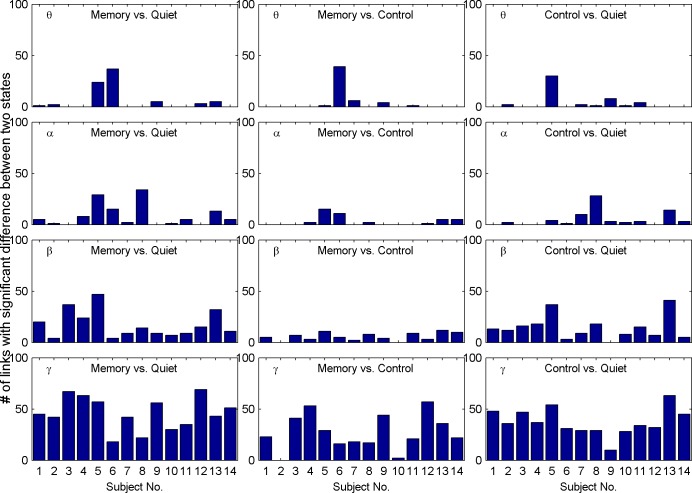
Statistics for the number of links with significant differences from state to state. From top to bottom they are theta, alpha, beta, and gamma, respectively, and from left to right they are memory vs. quiet, memory vs. control, and control vs. quiet, respectively. Within each sub figure, the horizontal axis represents the subject number, and the vertical axis represents the number of links with significant differences between the two states. This shows that although the total number of possible undirected links is 120 for 16 nodes, there are much fewer links that present significant between-states differences for the theta and alpha rhythms. Further, it is difficult to find common links whose variation remains consistent across subjects in these two rhythms. For the beta rhythm, there are more links differentiating quiet from memory or control; however, it is still difficult to differentiate memory from control. Among all four rhythms, gamma stands out prominently in that more links present significant differences.

Unsurprisingly, there are differences between individuals. In Fig 6, we present statistical bar graphs for the number of links with significant variations in correlation between states, in which the horizontal axis represents the subject number and the vertical axis represents the number of links with significant differences (*p*<0.001).

**Fig 7:**
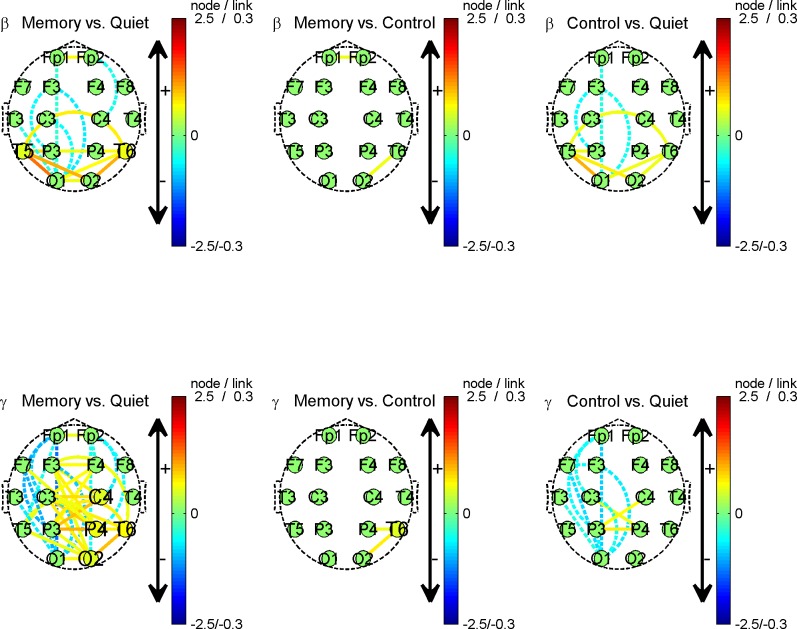
Brain connectivity variation topography for group average. Each row corresponds to a rhythm, and from top to bottom, they are beta and gamma, respectively; each column represents a comparison between states, and from left to right, they are memory vs. quiet, memory vs. control, and control vs. quiet, respectively. Lines between nodes depict the link strength variation, i.e. ∆*L*, between two states. A dashed line indicates a decreasing link strength and a solid line indicates an increasing link strength. The amplitude and sign of ∆*L* are also mapped into the color of the lines. Nodes (round patches) with captions represent the change of node connectivity, i.e., ∆*C*. In detail, a larger caption size means an increase in *C* and a smaller size means a decrease, while a medium size means no discernible changes. The amplitude and sign of ∆*C* are also mapped into the color of the node.

These results show that, although the total number of possible undirected links is 120 for 16 nodes, there are much fewer links that present significant between-states differences for the theta and alpha rhythms. Furthermore, it is difficult to find common links whose variation remains consistent across subjects in these two rhythms. For the beta rhythm, there are more links differentiating quiet from memory or control; however, it is still difficult to differentiate memory from control. Among all four rhythms, gamma stands out prominently in that more links present significant differences. Consequently, it is much easier to find common links with similar trans-state variation across subjects. For conciseness, we present group average results only for beta and gamma rhythms in Fig 7. Furthermore, subjects No. 6 and No. 13 are not taken into the average calculation because both had severely contaminated leads, as described in the section on EEG acquisition and preprocessing.

After group averaging, variations significant only for individual subject were suppressed, while the common variations in most subjects were kept. Therefore, as shown in Fig 7, is the results are not exactly the same as the individual result in Fig 5. Nevertheless, the most meaningful results were kept consistent:

1. In the beta rhythm, it was found that links between T5 and neighbor nodes P3, O1, O2, the symmetrical links between T6 and P4, O1, O2, and the link between T5 and T6 became stronger in the memory task compared with in the quiet state. The node connectivity of T5 and T6 also increased. A similar difference can be found in the comparison of control with quiet, though it is milder.
2. In the gamma rhythm, most ipsilateral frontal-posterior links become weaker while contra lateral links among Fp1, Fp2, F3, F4, C3, C4, P3, and P4 become stronger for the transition from quiet to memory, and this type of trend is maintained but milder when comparing control with quiet. There are much fewer differences between the control and memory states except that the node connectivity of T6 increases.

Considering the designed experimental scenes, these results convey certain meaningful information:

1. In memory and control scenes, subjects were confronted with objects combining shapes with colors. Perception of this type of visual stimuli involves an activity called a visual ventral stream, which starts from visual cortex V1 in the occipital lobe, then goes through V2 and V4 where shape and color can be differentiated, and ends in the inferior temporal cortex [22]. Considering the fact that areas of V1, V2, V4 and the inferior temporal cortex are close to the locations of O1/O2 and T5/T6, the strengthening of T5/T6-O1/O2 links in the beta rhythm may result from the ventral stream.
2. P3, P4, C3, C4, F3, F4, Fp1, and Fp2 are close to the cortex, which is involved in the visual dorsal stream [26], sensor and voluntary motion, visual attention and eye movements [27], and working memory [28]. The observation that contra lateral link strength among these locations increases implies that when being confronted with stimuli or tasks the contra lateral regions mentioned above interact more tightly in the gamma rhythm from the view of non-delayed linear correlation.
3. It is worth of noting that node connectivity increases in T6 when comparing the memory with control. In these two scenes, all visual stimuli and motors are similar; the only difference is the involvement of subjective mental effort to memorize or not. Considering that MTL might be needed in working memory tasks that requires binding and relational processing [2] and that the location of T6 is close to the MTL, the increase in T6 node connectivity may correspond to the subjective sensory information binding activity to inform memory.
4. As for significant ipsilateral frontal-posterior desynchronization for gamma in both memory and control, we give the reason of a sequential coordination manner. That is, according to neuroscience research, from posterior to frontal, the occipital and parietal cortex take responsibility of visual sensory processing, the super parietal cortex is associated with executive aspects of working memory and implementation of attention control, and the prefrontal cortex (PFC) is critical for resilient information maintenance [2]. This coordination is somewhat sequential rather than parallel in time. Therefore, when only a Pearson correlation with zero delay is considered, the real delay between channels will lead to a decreasing in the correlation coefficient.

In the results of representative subject of Fig 5, it is noted that the node connectivity of T4 increases instead of T6 when comparing memory and control. We attribute it to the individual difference as well as the blurring of spatial resolution in scalp EEG. In fact, both T4 and T6 are close to the underlying temporal lobe; therefore, it is reasonable to infer that the seemingly different phenomena have intrinsically originated from the same neural activities.

## Conclusion and Discussion

In this research, we present a comprehensive view for the brain coordination associated with WM for a relatively long time scale, extending beyond traditional stimuli lock averaging or restriction to short time scales of hundreds of milliseconds after stimuli onset. We first devised a calibration for the application of Pearson correlations in EEG analysis and then derived measures of link strength *L* and node connectivity *C*. Finally, we used these two measures to construct brain connectivity variation topography based on 16-lead scalp EEG associated with WM. It was found that the topography presents not only the difference brought about by visual stimuli and motor response, which can be observed between the quiet and memory states as well as between quiet and control, but also the difference brought about by subjective memorization, which is defined by the comparison of memory and control states. Specifically, the beta rhythm topography reveals the ventral stream of object visual pathway, and the gamma rhythm topography reflects the strengthening of contra lateral synchronization as well as the seemingly weakening of ipsilateral frontal-posterior synchronization, implying parallel inter-hemispheres coordination and sequential intra-hemisphere coordination during visual processing and motor control. Furthermore, gamma rhythm topography highlights the T6’s information binding and relational processing roles in memory forming through the comparison of the memory state with the control state. Considering the underlying neuroscience principles, our results are quite reasonable.

However, we failed to find consistent results at the group level for both the theta and alpha rhythms. As former research reported, when associated with WM, theta-band oscillations underlie the organization of sequentially ordered WM items, whereas alpha-band activity reflects the active inhibition of task-irrelevant information and attention allocation [2, 11], and they modulate high-frequency oscillations which directly take part in WM item encoding through phase-amplitude coupling (PAC), i.e., the amplitude of high frequency oscillations is modulated by the phase of a low-frequency rhythm [11]. This means that the phase correlation or synchronization in low-frequency oscillations between channels plays an essential role in WM. Thus, it is not difficult to understand that Pearson correlation, as an amplitude-amplitude correlation measure, fails to sensitively extract the coordination pattern in theta or alpha rhythm of scalp EEGs, which are themselves mixing signals of multiple underlying neural sources.

There are still several limitations in this work, including that only linear correlation without delay is taken into account. Coordination between brain regions involves various manners across multiple time scales, and it does take time to transmit neural signals. Therefore, in future work, the nonlinear correlation and time delay should be carefully studied. Although scalp EEG has the advantage of convenient acquisition, it has the problem of signal mixing [5]. We shall make more efforts to un-mix the signals to reconstruct the underlying sources for precise location in the future.

Nevertheless, the proposed brain connectivity variation topography based on continuous scalp EEG effectively captures brain connectivity variation associated with WM from relatively large scales both in time (from a second to a minute) and in space (from the scalp) for the first time. It can track brain activity continuously as it functions as usual with minimal manual interruption; therefore, we believe that it has promising potential for application in BCI and cognitive training.

## Supporting Information

S1 File EEG datasets with.mat format. (ZIP)
